# Supplementary material for: Magnetic field reveals vanishing Hall response in the normal state of stripe-ordered cuprates
Source: Nat Commun. 2021 Jun 17;12:3724. doi: 10.1038/s41467-021-24000-3 (PMC8211789; doi:10.1038/s41467-021-24000-3)
Supplement: Supplementary file 1 — Supplementary Information [file 41467_2021_24000_MOESM1_ESM.pdf]

Supplementary Information for Magnetic field reveals vanishing Hall response in the normal state of stripe-ordered cuprates

Zhenzhong Shi,[1,2] P. G. Baity,[1,3†] J. Terzic,[1] Bal K. Pokharel,[1,3]
T. Sasagawa,[4] Dragana Popović[1,3*]

[1]National High Magnetic Field Laboratory, Florida State University,
Tallahassee, Florida 32310, USA
[2]School of Physical Science and Technology & Institute for Advanced Study,
Soochow University, Suzhou 215006, China
[3]Department of Physics, Florida State University,
Tallahassee, Florida 32306, USA
[4]Materials and Structures Laboratory, Tokyo Institute of Technology,
Kanagawa 226-8503, Japan

[†] Present address: James Watt School of Engineering, University of Glasgow,
Glasgow, G12 8QQ, Scotland, United Kingdom
[*]To whom correspondence should be addressed; E-mail: dragana@magnet.fsu.edu

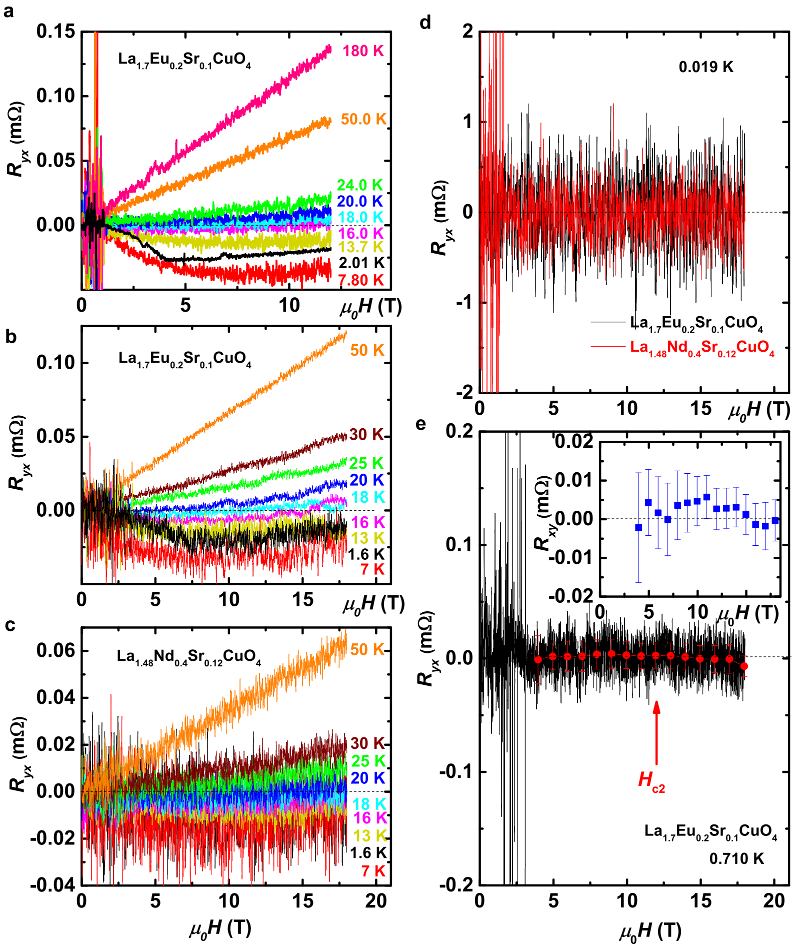

Supplementary Fig. 1: **Hall resistance R_{yx} vs H up to 18 T. a, b**, R_{yx} of $La_{1.7}Eu_{0.2}Sr_{0.1}CuO_4$, measured in 12 T and 18 T magnets, respectively, and **c**, R_{yx} of $La_{1.48}Nd_{0.4}Sr_{0.12}CuO_4$, measured up to 18 T, for several temperatures. At high T, $R_{yx} \propto H$, as expected in conventional metals. In the vortex solid phase where $\rho_{xx} = 0$, the Hall resistivity $\rho_{yx} = 0$, as expected for a superconductor (see, e.g., the 2.01 K trace in (**a**), for which Fig. 1 shows that the vortex solid melting field is ≈ 0.7 T). **d**, $R_{yx} = 0$ in $La_{1.7}Eu_{0.2}Sr_{0.1}CuO_4$ and $La_{1.48}Nd_{0.4}Sr_{0.12}CuO_4$ up to 18 T at the lowest $T = 0.019$ K, at which fields between the quantum melting field of the vortex solid (~ 5.5 T and ~ 4 T, respectively, for $La_{1.7}Eu_{0.2}Sr_{0.1}CuO_4$ and $La_{1.48}Nd_{0.4}Sr_{0.12}CuO_4$) and 18 T correspond to the viscous VL regime. **e**, $R_{yx} = 0$ in $La_{1.7}Eu_{0.2}Sr_{0.1}CuO_4$ in both viscous VL region ($H < H_{peak} \approx H_{c2}$) and the normal state ($H > H_{c2}$) at $T = 0.710$ K. Black trace: raw data, red dots: data averaged over 1 T bins, with error bars corresponding to ± 1 SD of the data points within each bin. By keeping T stable to within ≈ 1 mK during positive and negative H sweeps, the measurement resolution was increased significantly. Inset: A further increase in the resolution was achieved by averaging five different sets of measurements in which T during all ten H sweeps were equal to within < 2 mK (see also Supplementary Fig. 4); $\Delta R_{yx} \approx 5 \times 10^{-6}$ Ω. Dashed lines mark $R_{yx} = 0$ in all panels.

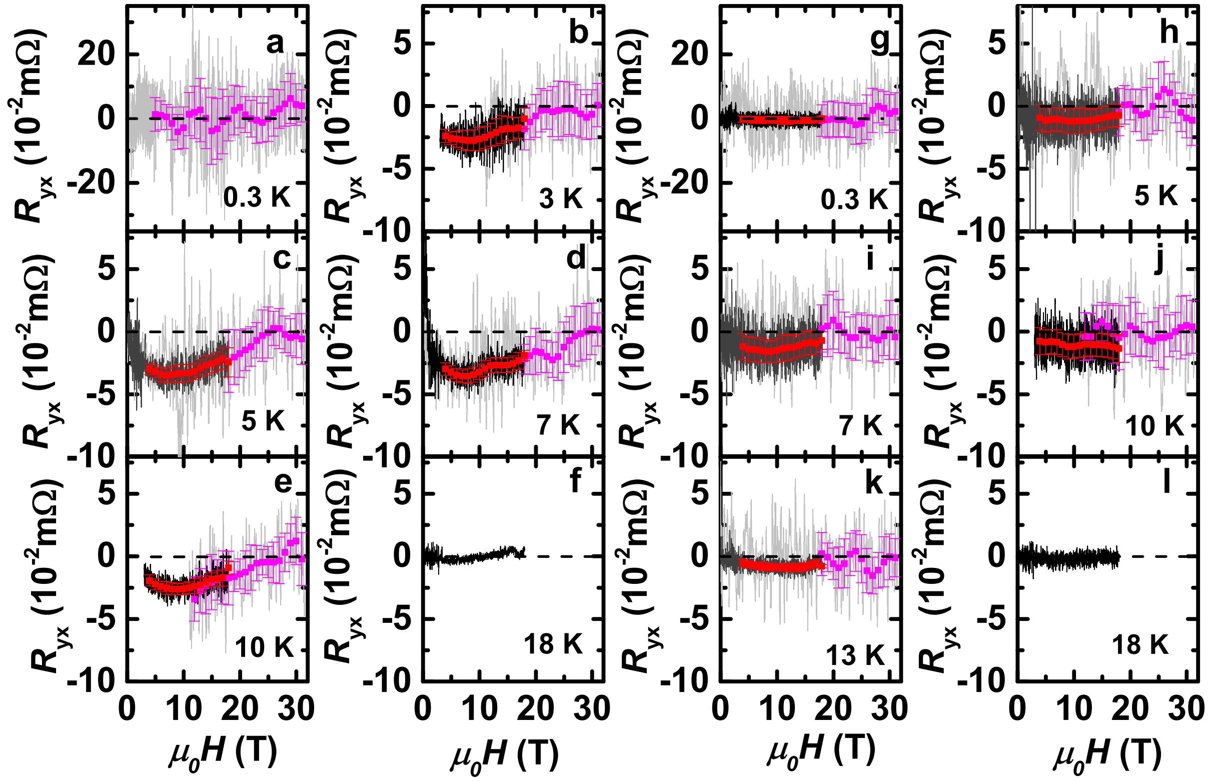

Supplementary Fig. 2: **Hall resistance R_{yx} vs H up to 31 T. a-f**, $La_{1.7}Eu_{0.2}Sr_{0.1}CuO_4$; **g-l**, $La_{1.48}Nd_{0.4}Sr_{0.12}CuO_4$. In all panels, black and grey traces represent the raw data obtained in two different runs with fields up to 18 T and 31 T, respectively. (The corresponding $R_H(H)$ data are shown in Supplementary Fig. 3.) The same R_{yx} data, averaged over 1 T bins, are shown by red and magenta symbols that correspond to the black and grey traces, respectively. Error bars correspond to 1 SD of the data points within each bin. At low T, the signals appear relatively noisy because extremely small excitation currents I are used to avoid heating and to ensure that the measurements are taken in the $I \to 0$ limit, since prior work has demonstrated[3] strongly nonlinear (i.e. non-Ohmic) transport in the presence of vortices. Dashed lines mark $R_{yx} = 0$.

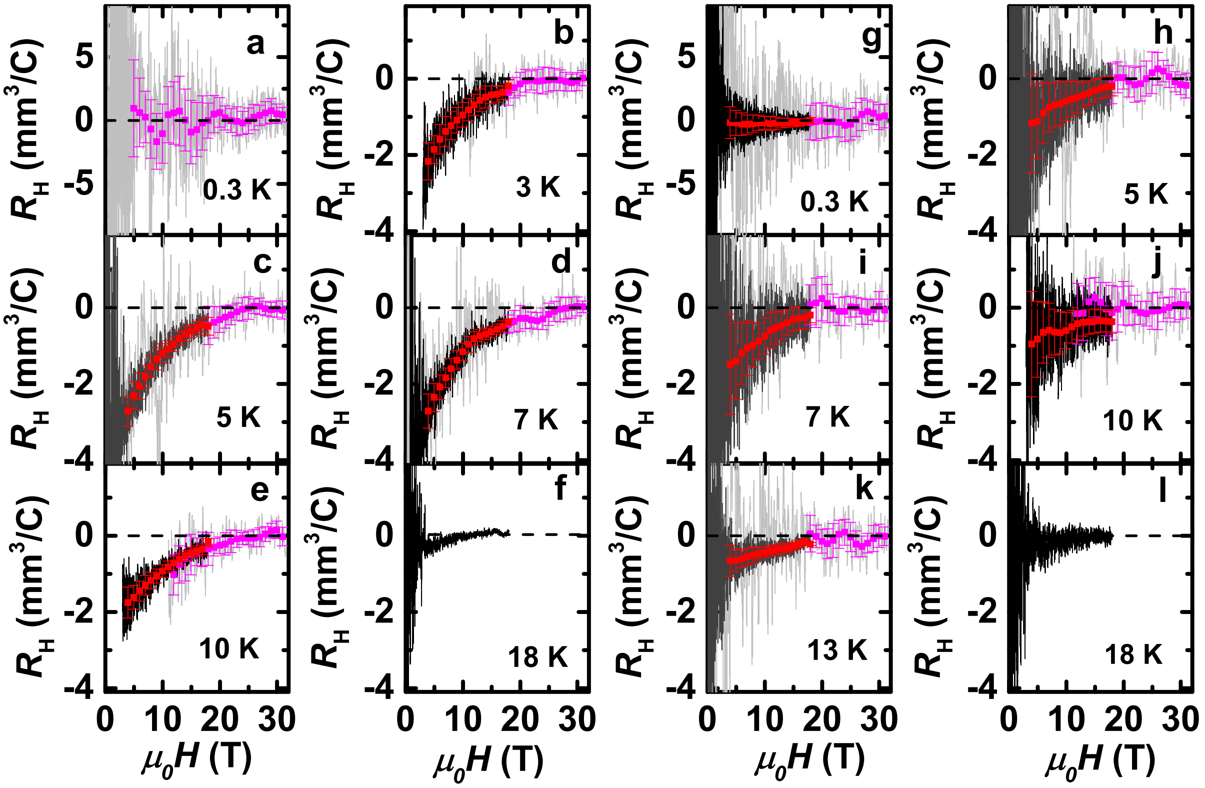

Supplementary Fig. 3: **Hall coefficient** $\boldsymbol{R_{\mathrm{H}}}$ **vs** $\boldsymbol{H}$ **up to 31 T. a-f**, $La_{1.7}Eu_{0.2}Sr_{0.1}CuO_4$; **g-l**, $La_{1.48}Nd_{0.4}Sr_{0.12}CuO_4$. In all panels, black and grey traces represent the raw data obtained in two different runs with fields up to 18 T and 31 T, respectively. (The corresponding $R_{yx}(H)$ data are shown in Supplementary Fig. 2.) The same R_{H} data, averaged over 1 T bins, are shown by red and magenta symbols that correspond to the black and grey traces, respectively. Error bars correspond to 1 SD of the data points within each bin. At low T, the signals appear relatively noisy because extremely small excitation currents I are used to avoid heating and to ensure that the measurements are taken in the $I \to 0$ limit, since prior work has demonstrated[12] strongly nonlinear (i.e. non-Ohmic) transport in the presence of vortices; at higher T, the error bars are 3-4 times smaller, $\Delta R_{\mathrm{H}} \sim 0.2 - 0.3$ mm^3/C, at the highest fields. Dashed lines mark $R_{\mathrm{H}} = 0$.

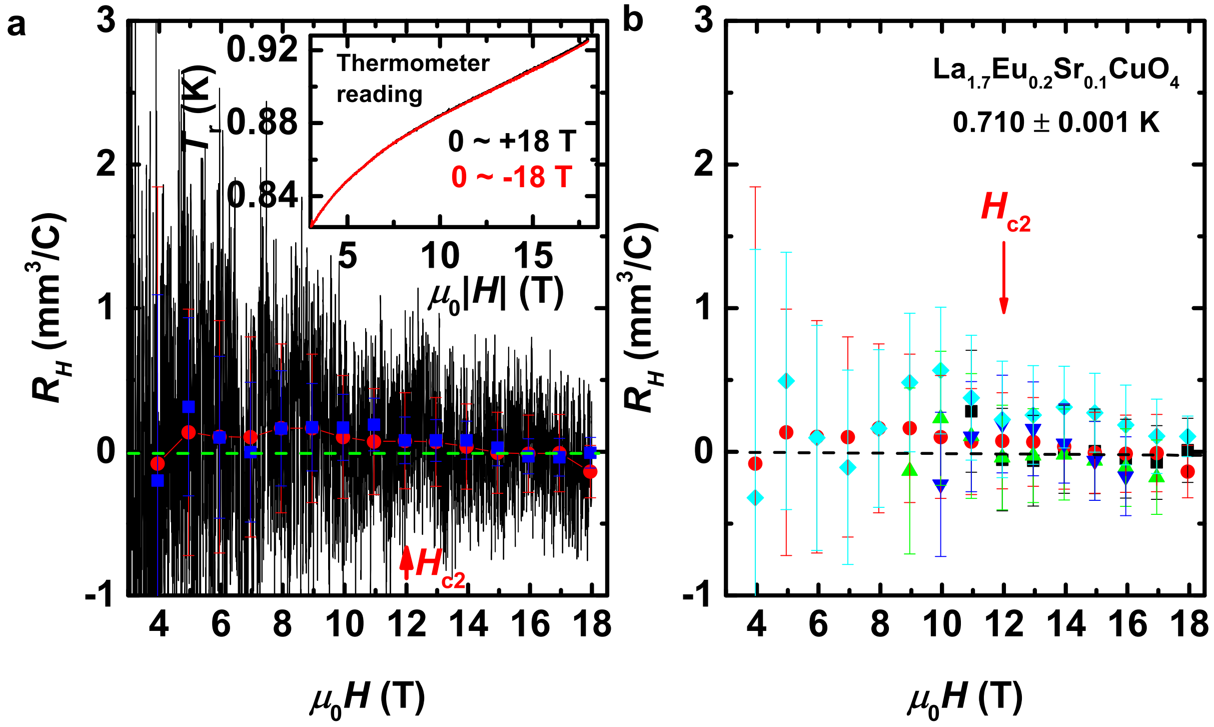

Supplementary Fig. 4: **Increasing the resolution of the Hall coefficient measurements in fields up to 18 T. a**, Black trace: the Hall coefficient $R_{\rm H}(H)$ in $La_{1.7}Eu_{0.2}Sr_{0.1}CuO_4$ corresponding to $R_{yx}(H)$ in Supplementary Fig. 1e; red dots: the same data averaged over 1 T bins, with error bars corresponding to ±1 SD of the data points within each bin. The inset shows the reading of the Cernox® thermometer, T_r, during positive and negative field sweeps. It should be noted that the H-dependence of T_r is dominated by the magnetoresistance of the thermometer and not by a change in the sample temperature (controlled by the sorb). The two $T_r(H)$ traces have practically identical profiles, corresponding to a T difference of at most $\approx$ 1 mK, which is crucial for these Hall measurements in which $R_{yx} \ll R_{xx}$. Blue dots in **a** represent $R_{\rm H}$ obtained by averaging over five different sets of measurements shown in **b**; blue dots thus correspond to $R_{yx}(H)$ in Supplementary Fig. 1e inset. Error bars in **b** correspond to 1 SD of the data points within each bin. A comparison of red and blue dots in **a** shows that, by averaging over five sets of measurements while keeping the temperature stable to within < 2 mK during ten H sweeps, the error was reduced from $\Delta R_{\rm H} \sim 0.2$ mm^3/C to $\Delta R_{\rm H} \sim 0.05$ mm^3/C (see also Supplementary Fig. 1e inset). In both panels, dashed lines mark $R_{\rm H} = 0$.

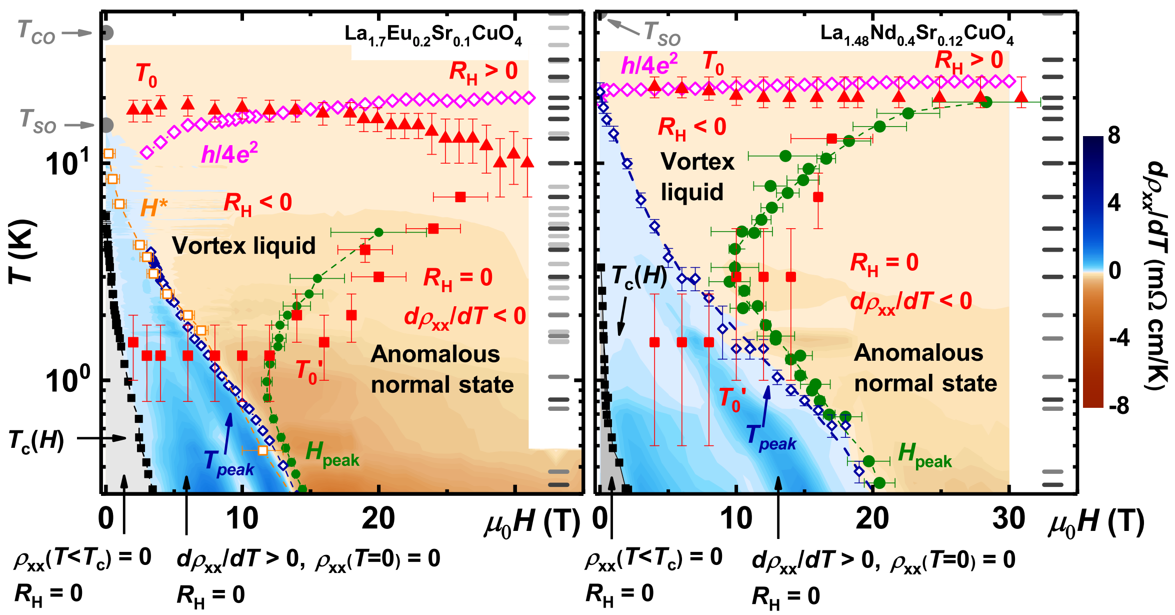

Supplementary Fig. 5: In-plane Hall coefficient R_H across the T–H phase diagram of striped cuprates. The phase diagram from Fig. 1, but here the color map shows slopes $d\rho_{xx}/dT$. The temperature dependence in the anomalous normal state is weak and insulating, i.e. $\rho_{xx} \propto \ln(1/T)$.

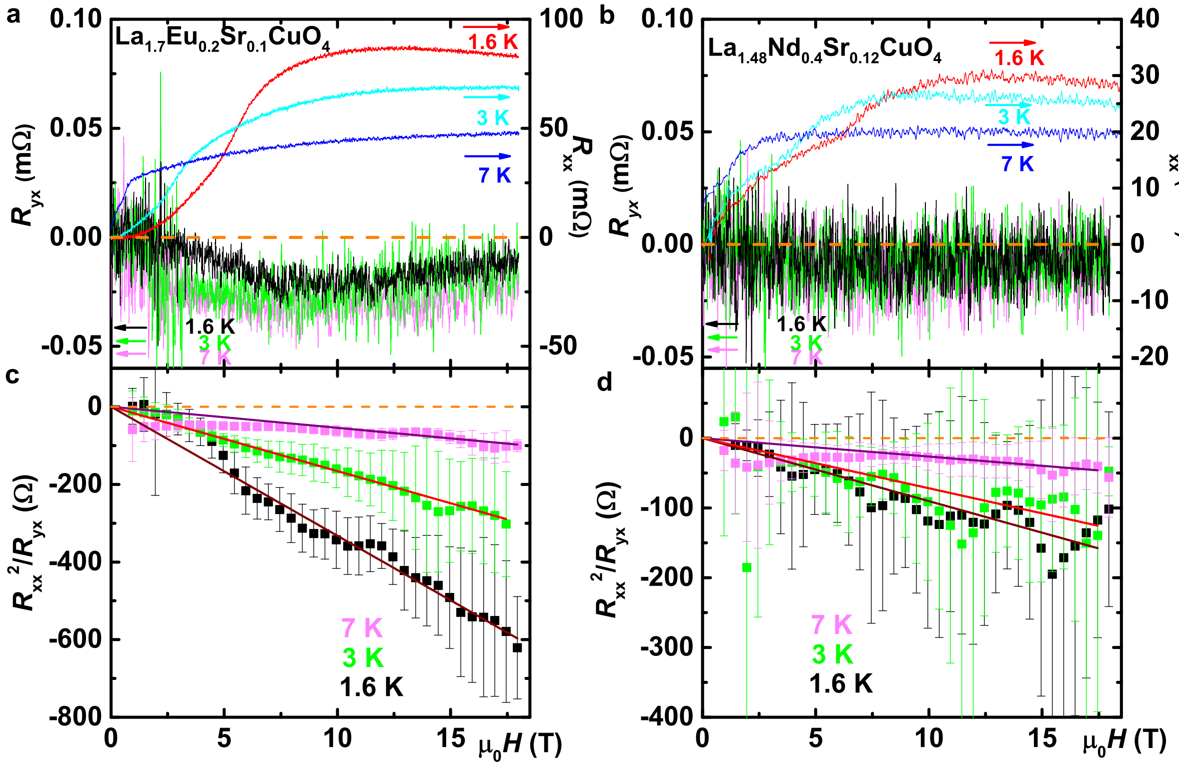

Supplementary Fig. 6: **Electrical transport in the vortex liquid regime. a** and **b**, Hall resistance R_{yx} (left axis) and longitudinal resistance R_{xx} (right axis) of $La_{1.7}Eu_{0.2}Sr_{0.1}CuO_4$ and $La_{1.48}Nd_{0.4}Sr_{0.12}CuO_4$, respectively, vs magnetic field at several temperatures within the vortex liquid regime. **c** and **d**, Scaling of the longitudinal and Hall resistance, ${\rho_{xx}}^2/\rho_{yx} \propto H$, for $La_{1.7}Eu_{0.2}Sr_{0.1}CuO_4$ and $La_{1.48}Nd_{0.4}Sr_{0.12}CuO_4$, respectively, at the same temperatures shown in **a** and **b**. R_{xx} and R_{yx} were averaged over 0.5 T bins before ${\rho_{xx}}^2/\rho_{yx}$ was calculated. Error bars correspond to 1 SD of the data points within each bin. Solid lines are linear fits going through the origin. The observed scaling indicates a state of dissipating vortex motion[35].
